# Outstanding Reviewers for *RSC Advances* in 2020

**DOI:** 10.1039/d1ra90103f

**Published:** 2021-05-24

## Abstract

We would like to take this opportunity to highlight the Outstanding Reviewers for *RSC Advances* in 2020, as selected by the editorial team for their significant contribution to the journal.
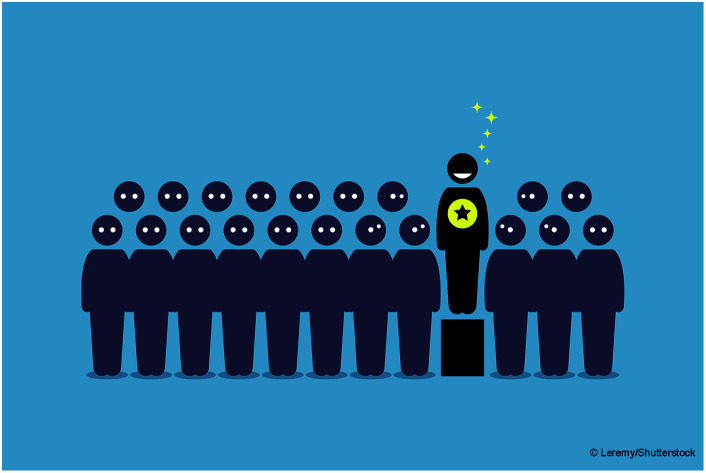

We would like to take this opportunity to thank all of *RSC Advances’* reviewers, and in particular highlight the Outstanding Reviewers for the journal in 2020, as selected by the editorial team for their significant contribution to *RSC Advances*. We would also like to direct a special thanks to the members of the *RSC Advances* Reviewer Panel for their hard work and dedication, and the valuable contribution they have made to the journal. The Reviewer Panel is a key part of our commitment to deliver rigorous and fair peer review and ensures that manuscripts are handled by experts throughout the peer review process. We are proud to work with these individuals and recognise their crucial role for the journal.

We announce our Outstanding Reviewers annually and each receives a certificate to give recognition for their contribution. The reviewers have been chosen based on the number, timeliness and quality of the reports completed over the year.

 

***RSC Advances* 2020 Outstanding Reviewers:**

 

Dr Amirul Abu Bakar

Universiti Malaysia Perlis

ORCID: 0000-0003-0579-552X

 

Dr Yuejie Ai

North China Electric Power University

ORCID: 0000-0001-6724-0971

 

Dr Thirumurugan Alagarsamy

Indian Institute of Science Education

ORCID: 0000-0001-8469-2718

 

Dr Federico Bella

Politecnico di Torino

ORCID: 0000-0002-2282-9667

 

Dr Satyapriya Bhandari

University of North Bengal

ORCID: 0000-0002-9150-6212

 

Dr Varsha Brahmkhatri

Jain University

ORCID: 0000-0002-9650-729X

 

Dr Manuel Cano

Universidad de Cordoba Facultad de Ciencias

ORCID: 0000-0002-0810-2920

 

Dr Gregory Caputo

Rowan University

ORCID: 0000-0002-4510-2815

 

Dr Ester Chiessi

Universita degli Studi di Roma Tor Vergata

ORCID: 0000-0001-7529-2755

 

Professor Beelee Chua

Korea University

ORCID: 0000-0002-9153-0167

 

Dr Anwang Dong

Beijing Institute of Technology

ORCID: 0000-0001-9068-6015

 

Professor Charles Gauthier

Institut National de la Recherche Scientifique

ORCID: 0000-0002-2475-2050

 

Dr Shiva Krishna Reddy Guduru

Baylor College of Medicine

ORCID: 0000-0002-6283-2908

 

Professor Ivanka Karadzic

Univerzitet u Beogradu

ORCID: 0000-0003-4694-7972

 

Dr Giuseppe Lazzara

University of Palermo

ORCID: 0000-0003-1953-5817

 

Dr Zhao Li

Wilmington PharmaTech

ORCID: 0000-0002-1119-6278

 

Dr Jinyang Li

Southwest Jiaotong University

ORCID: 0000-0001-8344-6494

 

Professor Sangbin Lim

Jeju National University

ORCID: 0000-0003-3653-5034

 

Dr Zhenghui Liu

Taizhou University

ORCID: 0000-0003-0559-4517

 

Dr Biswanath Mahanty

Karunya Institute of Technology and Sciences

ORCID: 0000-0002-5815-2440

 

Dr Navendu Mondal

The University of Texas at Dallas

ORCID: 0000-0001-5002-9678

 

Dr Adrian Nightingale

University of Southampton

ORCID: 0000-0003-2445-4827

 

Dr Bishnu Regmi

Florida Agricultural and Mechanical University

ORCID: 0000-0001-7690-9825

 

Professor Tiago Silva

Universidade Federal de Viçosa

ORCID: 0000-0002-7202-789X

 

Dr Tetsuro Soejima

Kinki Daigaku

ORCID: 0000-0003-2351-5616

 

Dr Shue Wang

University of New Haven

ORCID: 0000-0001-7382-5378

 

Dr Yuhao Wang

Stevens Institute of Technology

ORCID: 0000-0002-3974-0976

 

Dr Yan Xu

Northeastern University

ORCID: 0000-0002-7608-7013

 

Professor Yuanjian Zhang

Southeast University

ORCID: 0000-0003-2932-4159

 

Professor Mingshan Zhu

Jinan University

ORCID: 0000-0002-5926-5383

 

***RSC Advances* Reviewer Panel 2020 Outstanding Reviewers:**

 

Dr Vipul Agarwal

The University of New South Wales

ORCID: 0000-0002-6239-5410

 

Professor Abdullah Al-Mayouf

King Saud University

ORCID: 0000-0001-9246-7684

 

Dr María Báez

Universidad de Chile

ORCID: 0000-0003-0312-7237

 

Dr Rok Borštnar

National Institute of Chemistry Ljubljana

ORCID: 0000-0002-6786-5434

 

Professor Lingxin Chen

Yantai Institute of Coastal Zone Research, CAS

ORCID: 0000-0002-3764-3515

 

Dr Agustín G. Crevillén

Universidad Nacional de Educación a Distancia (UNED)

ORCID: 0000-0002-4470-6502

 

Professor Cleocir Dalmaschio

Federal University of Espírito Santo

ORCID: 0000-0002-3773-5786

 

Dr Narendar Reddy Gade

University of Alberta

ORCID: 0000-0002-6786-2604

 

Dr Darrick Heyd

Ryerson University

 

Dr Nesrin Horzum

İzmir Katip Çelebi University

ORCID: 0000-0002-2782-0581

 

Dr Yoshio Inagaki

The Society of Photography and Imaging of Japan

ORCID: 0000-0001-6397-1822

 

Dr Navendu Jana

AbbVie Inc.

ORCID: 0000-0002-7356-5382

 

Professor Beatriz Jurado Sánchez

Universidad de Alcalá

ORCID: 0000-0002-6584-1949

 

Professor Igor Komarov

National University of Kyiv

ORCID: 0000-0002-7908-9145

 

Dr S. Girish Kumar

CMR University

ORCID: 0000-0001-9132-1202

 

Professor Guijun Li

The Hong Kong University of Science and Technology

ORCID: 0000-0001-6259-3209

 

Professor Ekkehard Lindner

Universitat Tubingen

 

Dr Xin Liu

Dalian University of Technology

ORCID: 0000-0002-4422-4108

 

Dr Jinjie Qian

Wenzhou University

ORCID: 0000-0002-9996-7929

 

Dr Shiwei Qu

Scripps Research Institute

ORCID: 0000-0002-9358-066X

 

Dr Dharmarajan Rajarathnam

The University of Newcastle

ORCID: 0000-0001-6863-5510

 

Dr Leo Small

Sandia National Laboratories

ORCID: 0000-0003-0404-6287

 

Dr Lin Tang

Hunan University

ORCID: 0000-0001-6996-7955

 

Professor David Thompson

Sam Houston State University

ORCID: 0000-0002-2934-5729

 

Dr Paul Trippier

University of Nebraska Medical Center

ORCID: 0000-0002-4947-5782

 

Dr Nghia Truong Phuoc

Monash University

ORCID: 0000-0001-9900-2644

 

Dr Mark Waterland

Massey University

ORCID: 0000-0002-8493-9407

 

Professor Chunping Yang

Guangdong University of Petrochemical Technology

ORCID: 0000-0003-3987-2722

 

Professor Lei Yu

Yangzhou University

ORCID: 0000-0001-5659-7289

 

We would also like to thank the *RSC Advances* Editorial Board, Advisory Board and the research community for their continued support of the journal, as authors, reviewers and readers.

 

Laura Fisher, Executive Editor

Russell Cox, Editor-in-Chief

## Supplementary Material

